# Robotic radical nephrectomy and level II inferior vena cava thrombectomy: exploring the newer frontiers

**DOI:** 10.1590/S1677-5538.IBJU.2019.0614

**Published:** 2020-11-18

**Authors:** Puneet Ahluwalia, Ashwin Tamhankar, Gagan Gautam

**Affiliations:** 1 Max Institute of Cancer Care Department of Urooncology and Surgical Oncology New Delhi India Department of Urooncology and Surgical Oncology, Max Institute of Cancer Care, New Delhi, India; 2 Tata Memorial Hospital Department of Urooncology Mumbai India Department of Urooncology, Tata Memorial Hospital, Mumbai, India

## Abstract

**Objective:**

Renal tumors involving the inferior vena cava (IVC) appear to be a limitation to the application of minimal invasive surgery. Objective is to describe our technique in a patient undergoing robot-assisted radical nephrectomy (RARN) with level II IVC thrombectomy.

**Materials and Methods:**

Our index case is a 46-years old gentleman presenting with hematuria with 9.6 x 6.7cm mass in upper and mid pole of right kidney with level II IVC thrombus with cephalad extent of three centimeters beyond renal ostium. Vascular control was obtained with complete cross-clamping of the IVC by robotic bulldog clamps. The tumor thrombus was retrieved along with the IVC cuff as it was seen adherent to the IVC wall.

**Results:**

Patient successfully underwent right robotic radical nephrectomy with IVC thrombectomy without open conversion. Console time was 270 minutes with estimated blood loss of 300ml. No drain was placed and Foley's catheter was removed on POD 1. Patient was discharged on POD 3. Histopathology was suggestive of conventional clear cell carcinoma grade 3 with negative surgical margins. Follow-up at 6 months showed no evidence of recurrence.

**Conclusion:**

Robotic radical nephrectomy in the setting of IVC thrombus is feasible and can be performed safely in selected patients. Despite the complex and critical nature of these procedures, favorable outcomes and reproducibility can be expected with adequate robotic experience.

